# Impact of chest wall deformity on cardiac function by CMR and feature-tracking strain analysis in paediatric patients with Marfan syndrome

**DOI:** 10.1007/s00330-020-07616-9

**Published:** 2020-12-23

**Authors:** Hy Van Lam, Michael Groth, Thomas Mir, Peter Bannas, Gunnar K. Lund, Charlotte M. Jahnke, Malte Warncke, Kai-Jonathan Maas, Gerhard Adam, Jochen Herrmann, Enver Tahir

**Affiliations:** 1 Department of Pediatric Radiology, University Hospital Hamburg Eppendorf, Hamburg, Germany; 2 Department of Pediatric Cardiology, University Heart Center, Hamburg, Germany; 3 Department of Diagnostic and Interventional Radiology and Nuclear Medicine, University Hospital Hamburg Eppendorf, Martinistr. 52, 20246 Hamburg, Germany; 4 Department of General and Interventional Cardiology, University Heart Center, Hamburg, Germany

**Keywords:** Marfan syndrome, Magnetic resonance imaging, cine, Cardiac imaging techniques, Funnel chest, Ventricular dysfunction

## Abstract

**Objectives:**

To evaluate systolic cardiac dysfunction in paediatric MFS patients with chest wall deformity using cardiac magnetic resonance (CMR) imaging and feature-tracking strain analysis.

**Methods:**

Forty paediatric MFS patients (16 ± 3 years, range 8−22 years) and 20 age-matched healthy controls (16 ± 4 years, range 11−24 years) were evaluated retrospectively. Biventricular function and volumes were determined using cine sequences. Feature-tracking CMR was used to assess global systolic longitudinal (GLS), circumferential (GCS) and radial strain (GRS). A dedicated balanced turbo field echo sequence was used to quantify chest wall deformity by measuring the Haller index (HI).

**Results:**

LV volumes and ejection fraction (EF) were similar in MFS patients and controls. There was a trend for lower right ventricular (RV) volume (75 ± 17 vs. 81 ± 10 ml/m^2^, *p* = 0.08), RV stroke volume (41 ± 12 vs. 50 ± 5 ml/m^2^, *p* < 0.001) and RVEF (55 ± 10 vs. 62 ± 6%, *p* < 0.01) in MFS patients. A subgroup of MFS patients had an increased HI compared to controls (4.6 ± 1.7 vs. 2.6 ± 0.3, *p* < 0.001). They demonstrated a reduced RVEF compared to MFS patients without chest wall deformity (50 ± 11% vs. 58 ± 8%, *p* = 0.01) and controls (*p* < 0.001). LV GLS was attenuated when HI ≥ 3.25 (- 16 ± 2 vs. - 18 ± 3%, *p =* 0.03), but not GCS and GRS. LV GLS (*p <* 0.01) and GCS (*p* < 0.0001) were attenuated in MFS patients compared to controls, but not GRS (*p* = 0.31). RV GLS was attenuated in MFS patients compared to controls (- 21 ± 3 vs. - 23 ± 3%, *p* < 0.05).

**Conclusion:**

Chest wall deformity in paediatric MFS patients is associated with reduced RV volume, ejection fraction and GLS. Feature-tracking CMR also indicates impairment of systolic LV function in paediatric MFS patients.

**Key Points:**

*• Paediatric Marfan patients demonstrate reduced RV volume and ejection fraction compared to healthy controls.*

*• A concordant attenuation in RV global longitudinal strain was observed in Marfan patients, while the RV global circumferential strain was increased, indicating a possible compensatory mechanism.*

*• Subgroup analyses demonstrated alterations in RV ejection fraction and RV/LV global strain parameters, indicating a possible association of severe chest wall deformity with biventricular dysfunction in paediatric Marfan patients.*

**Supplementary Information:**

The online version contains supplementary material available at 10.1007/s00330-020-07616-9.

## Introduction

Marfan syndrome (MFS) is a heritable systemic connective tissue disorder, with a prevalence of 2−3:10,000 [1]. It is caused by mutations in the FBN1 gene, which encodes fibrillin-1, an important glycoprotein for the extracellular matrix [1]. The pleotropic effects of the disorder are seen primary in but are not limited to the ocular, skeletal and cardiovascular systems [2]. Pectus excavatum (PE) is one of the prominent skeletal disorders and up to 70% of patients with MFS are reported to have PE [3]. The well-known cardiovascular manifestations are aortic dilatation and dissection, and heart failure caused by aortic or mitral valve regurgitation [2, 4]. However, there are studies suggesting the existence of cardiac dysfunction in the absence of valvular disease [5–9].

Previous studies assessing the ventricular dimensions and function in MFS patients report discordant results. While Meijboom et al and Chatrath et al could identify dilatation of the left ventricle (LV) in some of their patients [10, 11], volumes, mass and function were normal in the study by Savolainen et al [12]. More recent studies using advanced echocardiography and cardiac magnetic resonance (CMR) identified distinct systolic and diastolic dysfunction in patients with MFS [5–7, 13, 14]. However, the possible effects of a chest wall deformity on cardiac morphology and function in MFS patients have not been studied yet. There are numerous studies investigating the effect of PE on the heart in patients without MFS [15–20]. While results are inconclusive, some suggest that surgery provides decompression of the cardiac structures and improvement of function, particularly of the right ventricle (RV) [16–18].

To the best of our knowledge, there are no studies reporting the effect of chest wall deformity on cardiac function in patients with MFS. The purpose of our study was to evaluate systolic cardiac dysfunction in paediatric MFS patients with chest wall deformity using CMR imaging and feature-tracking strain analysis.

## Materials and methods

### Study population

**Fig. 1:**
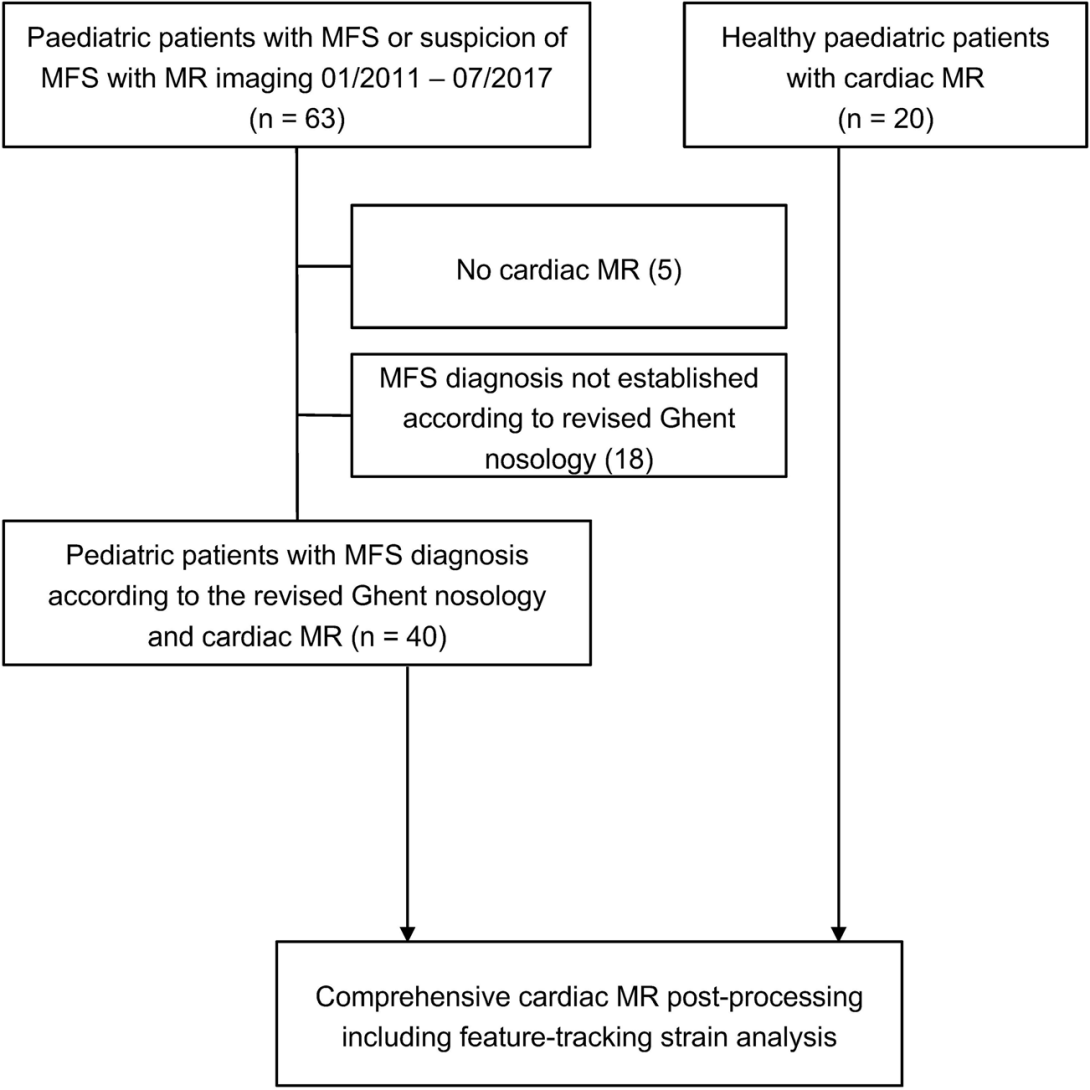
Flow chart showing the acquisition of the study population, consisting of a paediatric patient group with MFS and of a healthy control group

The local clinical institutional review board approved our retrospective study and waived the requirement for informed consent due to the anonymous analysis of all data. The medical image archive of our institution was reviewed for paediatric patients with the established diagnosis of MFS according to the revised Ghent nosology [21], who had undergone CMR imaging between January 2011 and July 2017. Forty paediatric patients (aged 16 ± 3 years, range 8−22 years) fulfilled the inclusion criteria (Fig. 1). An age-matched group of 20 healthy individuals (aged 16 ± 4 years, range 11−24 years) was included as a control group, who were initially referred to our institution with suspicion of arrhythmogenic right ventricular dysplasia or viral myocarditis, but were finally cleared of all suspicion for cardiovascular diseases. To the best of our knowledge, the control subjects had no other underlying medical conditions.

### CMR protocol

Studies were performed on a 1.5-T Achieva scanner with a 5-channel cardiac phased array receiver coil (Phillips, Healthcare). ECG-triggered steady-state free-precession (SSFP) cine sequences were acquired in short axis and 2-, 3- and 4-chamber views. The following typical imaging parameters were used for cine series: acquired voxel size, 1.98 × 1.80 × 6 mm^3^; reconstructed voxel size, 1.36 × 1.36 × 6 mm^3^; gap, 4 mm; 9–10 slices for full LV coverage; echo time, 1.67 ms; time to repetition, 3.34 ms; flip angle, 60°; sense factor, 2.0; 25 phases per RR interval.

In addition, images dedicated for the evaluation of chest wall deformities were acquired as ECG-triggered balanced turbo field echo (BTFE) on the transversal plane with the following imaging parameters: acquired voxel size, 1.68 × 1.67 × 10 mm^3^; reconstructed voxel size, 1.41 × 1.41 × 10 mm^3^; gap, 5.10 mm; echo time, 1.53 ms; time to repetition, 3.06 ms; flip angle, 90°.

### Biventricular and biatrial morphology and function quantification

Cardiac volumes and myocardial mass were determined with CVi42 (Circle Cardiovascular Imaging Inc.). Endocardial contours were manually traced in end-diastole and end-systole on short-axis cine images to calculate end-systolic (ESV) and end-diastolic volumes (EDV) as well as stroke volume (SV) and ejection fraction (EF) [22, 23]. Epicardial contours were traced to measure LV mass [22]. By tracing the endocardial contours in 4- and 2-chamber long-axis cine images, volumetric parameters of the left atrium (LA) were measured, while for the right atrium (RA), only the 4-chamber cine images were used [24].

### LV and RV global strain analysis

**Fig. 2:**
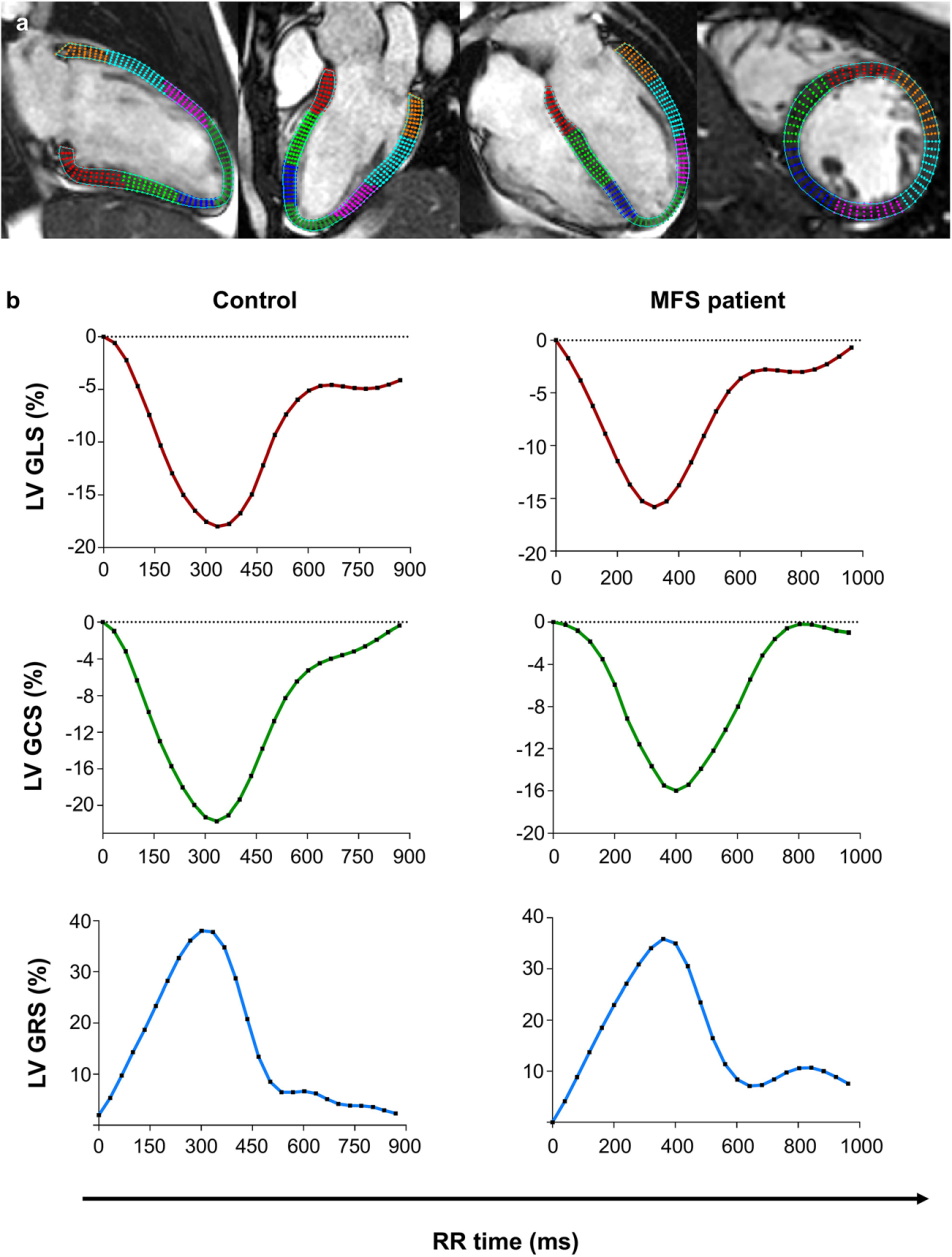
Comparison of LV global longitudinal (GLS), circumferential (GCS) and radial strain (GRS) in a healthy control and a MFS patient. Strain values were determined by feature-tracking CMR using software, which automatically propagates the manually drawn epi- and endocardial contours through all cardiac phases of the 2-, 3- and 4-chamber and short-axis cine series of a MFS patient (**a**). Global strain values of a healthy control and a MFS patient are depicted (**b**)

Global peak systolic strain was quantified using the software Segment (Medviso, Version 2.1.R.6108) by contouring endo- and epicardial borders in end-diastole. Contours were then automatically propagated throughout all cardiac phases to calculate strain values (Fig. 2) [25]. Long-axis cine images were used to calculate LV global longitudinal (GLS) and radial strain (GRS) as previously described [26]. LV global circumferential strain (GCS) was determined using basal, mid and apical short-axis cine slices [26]. Long-axis 4-chamber and three short-axis series were used to calculate RV GLS and RV GCS respectively, by contouring the RV endocardium [25].

### Quantification of chest wall deformity

**Fig. 3:**
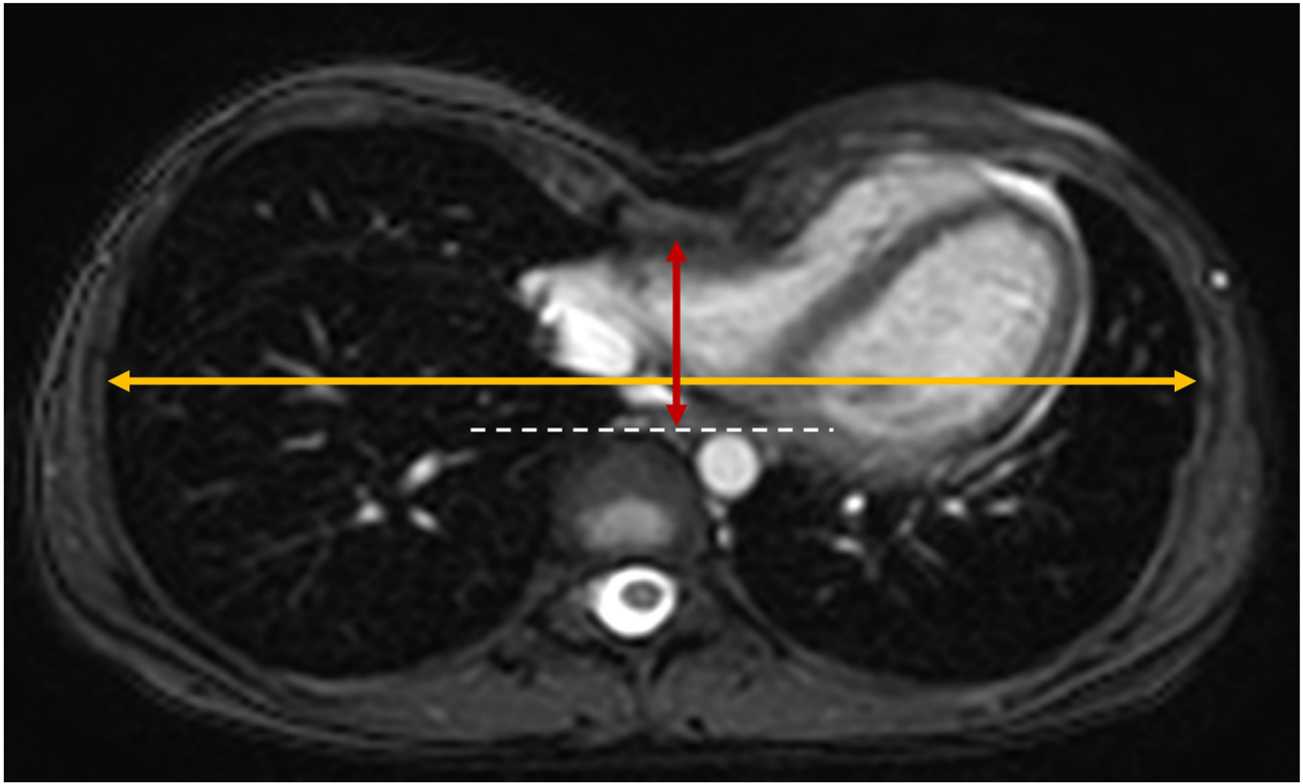
Measurement technique for the estimation of the Haller index. Transversal CMR images (BTFE) of a MFS patient were used to evaluate the severity of a pectus excavatum. Haller index was estimated as the ratio of the maximum transverse diameter of the chest wall (yellow line) to the minimum sternovertebral diameter (red line). This patient showed a severe pectus excavatum with a Haller index of 4.5 as well as a compression of the right ventricle

Chest wall deformity was quantified using the severity of pectus excavatum in patients with MFS by measuring the Haller index (HI) on a BTFE sequence. HI was calculated as the ratio of the maximum transverse diameter of the chest wall to the minimum sternovertebral diameter (Fig. 3) [27].

### Subgroup analysis of patients with chest wall deformity

To further assess the influence of a chest wall deformity on cardiac function, we stratified MFS patients using a cut-off for HI of 3.25 or greater. This cut-off is considered to indicate a severe chest wall deformity and surgical correction is recommended [28].

### Statistical analysis

Statistical analyses were performed with IBM SPSS Statistics 25 (IBM, Version 25.0). Homogeneity of variance was tested with the Levene test. When homogeneity of variance was given, the mean differences between groups were compared with the independent sample *t* test, if not the Welch test was used. Categorical variables were compared using the chi^2^ test or Fisher’s exact test as appropriate. Continuous variables are shown as mean ± SD and two-sided *p* values < 0.05 were considered significant.

## Results

### Demographics of MFS patients compared to controls

Demographics of MFS patients and controls are shown in Table 1. The mean age in both groups was 16 years. MFS patients were significantly taller than the control group (1.79 ± 0.16 vs. 1.70 ± 0.12 m, *p* = 0.03), while having similar weight (62 ± 20 vs. 61 ± 15 kg, *p* = 0.90), resulting in a significantly lower BMI (19 ± 4 vs. 21 ± 3 kg/m^2^, *p* < 0.05). BSA did not show any differences (*p* = 0.44). MFS patients had a significantly higher HI (3.4 ± 1.5 vs 2.6 ± 0.3, *p* < 0.01).

**Table 1:** Comparison of MFS patients with healthy controls

	Controls (*n* = 20)	All patients (*n* = 40)	*p* value
Demographics			
Age, years	16 ± 4	16 ± 3	0.77
Male, %	12	23	> 0.99
Weight, kg	61 ± 15	62 ± 20	0.90
Height, m	1.70 ± 0.12	1.79 ± 0.16	0.03
BMI, kg/m^2^	21 ± 3	19 ± 4	< 0.05
BSA, m^2^	1.70 ± 0.26	1.77 ± 0.35	0.44
CMR parameters			
Heart rate at CMR, bpm	73 ± 14	71 ± 11	0.50
LVEF, %	62 ± 5	61 ± 5	0.66
LV mass, g/m^2^	54 ± 10	55 ± 12	0.85
LVEDV, ml/m^2^	82 ± 12	86 ± 19	0.38
LVESV, ml/m^2^	31 ± 8	33 ± 8	0.56
LVSV, ml/m^2^	50 ± 7	51 ± 11	0.62
LAEDV, ml/m^2^	11 ± 3	14 ± 7	0.05
LAESV, ml/m^2^	29 ± 8	28 ± 11	0.65
RVEF, %	62 ± 6	55 ± 10	< 0.01
RVEDV, ml/m^2^	81 ± 10	75 ± 17	0.08
RVESV, ml/m^2^	32 ± 7	34 ± 10	0.35
RVSV, ml/m^2^	50 ± 5	41 ± 12	< 0.001
RAEDV, ml/m^2^	20 ± 8	19 ± 7	0.42
RAESV, ml/m^2^	34 ± 9	33 ± 10	0.68
Chest wall dimensions			
A-Haller, mm	229 ± 17	227 ± 23	0.81
C-Haller, mm	89 ± 13	77 ± 25	0.02
Haller index	2.6 ± 0.3	3.4 ± 1.5	< 0.01
Global LV myocardial strain			
LV GLS, %	− 20 ± 3	− 17 ± 3	< 0.01
LV GCS, %	− 22 ± 3	− 19 ± 3	< 0.0001
LV GRS, %	37 ± 8	35 ± 7	0.31
Global RV myocardial strain			
RV GLS, %	− 23 ± 3	− 21 ± 3	< 0.05
RV GCS, %	− 12 ± 3	− 15 ± 3	< 0.01

Numbers are mean ± SD for continuous and *n* (%) for categorical data

*Abbreviations*: *BMI*, body mass index; *BSA*, body surface area; *GCS*, global circumferential strain; *GLS*, global longitudinal strain; *GRS*, global radial strain; *HR*, heart rate; *LAEDV*, left atrial end-diastolic volume; *LAESV*, left atrial end-systolic volume; *LV*, left ventricular; *LVEF*, left ventricular ejection fraction; *LVEDV*, left ventricular end-diastolic volume; *LVESV*, left ventricular end-systolic volume; *LVSV*, left ventricular stroke volume; *RAEDV*, right atrial end-diastolic volume; *RAESV*, right atrial end-systolic volume; *RV*, right ventricle; *RVEDV*, right ventricular end-diastolic volume; *RVEF*, right ventricular ejection fraction; *RVESV*, right ventricular end-systolic volume; *RVSV*, right ventricular stroke volume

### Cardiac morphology and function of MFS patients compared to controls

Cardiac function parameters are given in Table 1. LVEF was not different between MFS patients and controls (61 ± 5 vs. 62 ± 5%, *p* = 0.66). There were no significant differences in left ventricular volumes including LVEDV (86 ± 19 vs. 82 ± 12 ml/m^2^, *p* = 0.38) and LV mass (*p* = 0.85). RVEF was significantly lower in MFS patients compared to controls (55 ± 10 vs. 62 ± 6 %, *p* < 0.01). There was a trend for reduced RVEDV in MFS patients (75 ± 17 vs. 81 ± 10 ml/m^2^, *p* = 0.08). RVESV was not different (*p* = 0.35). RVSV was significantly lower in MFS patients compared to controls (41 ± 12 vs. 50 ± 5 ml/m^2^, *p* < 0.001). Using the cine series, valvular regurgitation was identified in 12 MFS patients (mild tricuspid (5), mild mitral (6) and mild aortic regurgitation (2)) and one MFS patient had mild tricuspid and aortic regurgitation. Ten controls also showed valvular regurgitation (mild tricuspid (8) and mild aortic regurgitation (2)). CMR characteristics of MFS patients with and without mild valvular regurgitation are given in Supplemental Table E1.

### Global myocardial strain in MFS patients compared to controls

LV GLS (- 17 ± 3 vs. - 20 ± 3%, *p* < 0.01) and LV GCS (- 19 ± 3 vs. - 22 ± 3%, *p* < 0.0001) were attenuated in MFS patients compared to controls (Table 1, Fig. 2). LV GRS (35 ± 7 vs. 37 ± 8%, *p* = 0.31) showed no significant differences between both groups (Table 1, Fig. 2). RV GLS was attenuated compared to controls (- 21 ± 3 vs. - 23 ± 3%, *p* < 0.05), while RV GCS was increased in MFS patients (- 15 ± 3 vs. - 12 ± 3%, *p* < 0.01) (Table 1).

### CMR characteristics of MFS patients with chest wall deformity

Table 2 gives the demographics and CMR characteristics of MFS patients stratified using a cut-off for HI of 3.25. Seventeen MFS patients had a HI ≥ 3.25 and 23 MFS patients a HI < 3.25; the mean HI was 4.6 ± 1.7 and 2.5 ± 0.4 (*p* < 0.0001), respectively. A significant difference was seen in the sternovertebral diameter (54 ± 15 vs. 94 ± 15 mm, *p* < 0.0001), but not in the transverse diameter (*p* = 0.71). MFS patients with a HI ≥ 3.25 were younger (*p* = 0.04) and weighted less (*p* < 0.01). The height was not different (*p* = 0.11). BMI (*p* < 0.001) and BSA (*p* < 0.01) were both lower in the HI ≥ 3.25 group. LV volumes and mass showed no differences (Table 2). RVEF was reduced in the group with HI ≥ 3.25 (50 ± 11 vs. 58 ± 8%, *p* = 0.01), while there was a trend for increased RVESV (37 ± 12 vs. 32 ± 8 ml/m^2^, *p* = 0.09) and reduced RVSV (37 ± 12 vs. 44 ± 11 ml/m^2^, *p* = 0.08). LV GLS was attenuated when HI ≥ 3.25 (- 16 ± 2 vs. - 18 ± 3%, *p* = 0.03), but LV GCS and LV GRS were not significantly different between both groups. RV GLS and GCS were similar in both groups (Table 2).

**Table 2:** Comparisons of MFS patients with or without chest wall deformity and healthy controls

	Haller index < 3.25 (*n* = 23)	*p* value	Haller index ≥ 3.25 (*n* = 17)	*p* value	Controls (*n* = 20)
Demographics					
Age, years	17 ± 3	0.04	14 ± 3	0.23	16 ± 4
Male, %	14	0.75	9	0.75	12
Weight, kg	70 ± 20	< 0.01	51 ± 15	0.05	61 ± 15
Height, m	1.83 ± 0.16	0.11	1.74 ± 0.16	0.35	1.70 ± 0.12
BMI, kg/m^2^	21 ± 4	< 0.001	16 ± 3	< 0.0001	21 ± 3
BSA, m^2^	1.89 ± 0.33	< 0.01	1.61 ± 0.30	0.32	1.70 ± 0.26
CMR parameters					
HR at CMR, bpm	71 ± 13	0.89	71 ± 7	0.51	73 ± 14
LVEF, %	61 ± 5	0.98	61 ± 5	0.71	62 ± 5
LV mass, g/m^2^	55 ± 11	0.90	54 ± 14	0.94	54 ± 10
LVEDV, ml/m^2^	87 ± 17	0.76	85 ± 22	0.60	82 ± 12
LVESV, ml/m^2^	34 ± 8	0.38	31 ± 7	0.97	31 ± 8
LVSV, ml/m^2^	53 ± 12	0.23	49 ± 10	0.60	50 ± 7
LAEDV, ml/m^2^	16 ± 7	0.07	12 ± 6	0.64	11 ± 3
LAESV, ml/m^2^	31 ± 11	0.01	22 ± 8	0.02	29 ± 8
RVEF, %	58 ± 8	0.01	50 ± 11	< 0.001	62 ± 6
RVEDV, ml/m^2^	75 ± 16	0.93	75 ± 19	0.23	81 ± 10
RVESV, ml/m^2^	32 ± 8	0.09	37 ± 12	0.09	32 ± 7
RVSV, ml/m^2^	44 ± 11	0.08	37 ± 12	< 0.001	50 ± 5
RAEDV, ml/m^2^	20 ± 7	0.26	17 ± 6	0.21	20 ± 8
RAESV, ml/m^2^	36 ± 10	0.06	29 ± 10	0.14	34 ± 9
Chest wall dimensions					
A-Haller, mm	226 ± 20	0.71	229 ± 28	> 0.99	229 ± 17
C-Haller, mm	94 ± 15	< 0.0001	54 ± 15	< 0.0001	89 ± 13
Haller index	2.5 ± 0.4	< 0.0001	4.6 ± 1.7	< 0.001	2.6 ± 0.3
Global LV myocardial strain					
LV GLS, %	− 18 ± 3	0.03	− 16 ± 2	< 0.001	− 20 ± 3
LV GCS, %	− 19 ± 2	0.21	− 18 ± 4	< 0.001	− 22 ± 3
LV GRS, %	35 ± 8	0.50	34 ± 7	0.22	37 ± 8
Global RV myocardial strain					
RV GLS, %	− 22 ± 3	0.45	− 21 ± 2	< 0.05	− 23 ± 3
RV GCS, %	− 15 ± 4	0.73	− 14 ± 3	0.02	− 12 ± 3

Numbers are mean ± SD for continuous and *n* (%) for categorical data

### CMR characteristics of MFS patients with chest wall deformity compared to controls

MFS patients with HI ≥ 3.25 were compared to controls in Table 2. Age, weight, height and BSA showed no significant differences, but patients had a lower BMI (16 ± 3 vs. 21 ± 3 kg/m^2^, *p* < 0.0001). There were no differences in LV morphological and functional parameters. RVEF (50 ± 11 vs. 62 ± 6%, *p* < 0.001) and RVSV (37 ± 12 vs. 50 ± 5 ml/m^2^, *p* < 0.001) were decreased in patients with HI ≥ 3.25. There was a trend for increased RVESV in MFS patients with chest wall deformity (37 ± 12 vs. 32 ± 7 ml/m^2^, *P* = 0.09). LV GLS (- 16 ± 2 vs. - 20 ± 3%, *p* < 0.001) and LV GCS (- 18 ± 4 vs. - 22 ± 3%, *p* < 0.001) were attenuated in MFS patients with HI ≥ 3.25. LV GRS (*p* = 0.22) was not significantly different. RV GLS was attenuated (- 21 ± 2 vs. - 23 ± 3%, *p* < 0.05), while RV GCS was increased (- 14 ± 3 vs. - 12 ± 3%, *p* = 0.02) in patients with HI ≥ 3.25 (Table 2).

## Discussion

This study investigated the occurrence of morphological alterations and systolic dysfunction in paediatric patients with MFS by CMR and feature-tracking strain analysis compared to healthy controls. Subgroup analyses were used to assess the role of chest wall deformity in the attenuation of systolic myocardial function. The major findings are the following:(1) RVEF and RVSV were reduced in MFS patients and especially in those with severe chest wall deformity. (2) LV volumes, mass and function were similar between MFS patients and healthy controls. However, LV GLS and LV GCS were attenuated in MFS patients, suggesting subtle LV function alterations, which were only detectable by feature-tracking CMR analysis. (3) RV GLS was attenuated, and RV GCS was increased in MFS patients.

### LV and RV function in MFS patients

Early echocardiographic and CMR studies have demonstrated impairment of cardiac function in MFS patients without valvular diseases, which was interpreted as Marfan cardiomyopathy [5–8, 13, 14]. A CMR study by Alpendurada et al detected reduced LVEF and increased LV volumes in up to 25% of adult MFS patients [5]. De Backer et al also observed a reduction in LVEF and increased end-systolic LV volume in adult MFS patients using CMR [6]. In our study LVEF and volumes were similar between paediatric MFS patients and controls, which could be attributable to study population differences. It has been shown that mitral valve prolapse is present in up to 40% and severe forms might involve 12% of the adult MFS population [29], whereas paediatric MFS patients are less likely to be affected by valvular disease. In line with our findings, Savolainen et al analysed paediatric MFS patients without significant valvular disease using CMR and observed no differences in systolic LV function and volumes compared to controls [12]. Thus, a reduction in LVEF might not yet be prevalent in paediatric MFS patients and manifest later in adulthood. However, it might also indicate that LVEF is not sensitive enough to detect subtle changes in cardiac function in paediatric MFS patients, as has been previously reported for patients with heart failure with preserved ejection fraction [30, 31].

Only a few studies have reported a possible impairment of RV function in MFS patients [5, 7]. Alpendurada et al showed a mild reduction in RVEF in 10% of their patients [5]. Furthermore, De Witte et al demonstrated reduced RVEF compared to controls using CMR [7]. Concordantly, we observed a decreased RVEF in our MFS cohort. Additionally, in a subgroup analysis, we detected a significant reduction of RVEF in patients with severe chest wall deformity compared to patients with HI < 3.25. Thus, a dependence between the severity of chest wall deformity and the RV impairment in MFS patients can be postulated. Saleh et al investigated cardiac anatomy and function in non-MFS patients with severe PE using CMR [15]. Consistent with our results, a significant reduction of RVEF in patients with PE was found, while LVEF remained normal [15]. The authors also demonstrated a geometric RV distortion, which was attributed to sternal compression [15]. Topper et al went a step further and investigated cardiac function before and after surgical repair of PE [16], showing the influence of chest wall deformity on the cardiac function directly. The authors observed a decreased RVEF before and a significant improvement after thoracic surgery in 38 patients [16]. Even though both studies recruited non-MFS patients with more severe chest wall deformity, the consistency to our findings of attenuated RV function provides arguments for RV functional alterations.

### Strain analysis in patients with MFS

In our cohort of MFS patients, LVEF was similar to healthy controls. However, strain analysis by feature-tracking CMR showed an attenuation of LV global longitudinal and circumferential strain in MFS patients. Furthermore, MFS patients with severe chest wall deformity had a lower LV contractility than those with HI < 3.25 as indicated by attenuated LV global longitudinal strain. Studies reporting on a comprehensive strain analysis in MFS patients are scarce. Scherptong et al investigated 50 adult MFS patients and 50 controls using echocardiography and concordant to our findings LV longitudinal strain was significantly decreased in MFS patients, while LV circumferential strain and EF did not differ [32]*.* Kiotsekoglou et al used echocardiography and demonstrated reduced LV longitudinal and radial strain in adult MFS patients compared to controls [33]*.* Angtuaco et al did not observe any significant differences in LV longitudinal, radial and circumferential strain in a small cohort of paediatric MFS patients (*N* = 16) compared to controls, but detected reduced regional strain rates, suggesting that the study might be underpowered [34]*.* To date, there is only one retrospective CMR study by Winther et al that used feature-tracking CMR to characterise adult MFS patients, demonstrating a reduced LV longitudinal strain pattern in some patients [9]. However, the authors could not report on LV circumferential strain due to lacking short-axis cine stack in their CMR protocol [9]. The majority of studies seem to agree on a decrease of LV longitudinal strain in MFS patients, but are inconclusive regarding LV circumferential and radial strain. LV longitudinal strain is known to be more reproducible than LV circumferential and radial strain in echocardiography [35]. Also, other CMR studies have demonstrated the value of LV longitudinal strain for the detection of subtle cardiac impairment and for outcome prediction, for example in acute myocarditis or cardiac sarcoidosis [36, 37]. Our results support the notion that subtle LV systolic dysfunction in MFS patients exists, but cannot be detected by standard ejection fraction. A prospective longitudinal study would be warranted to investigate, whether early reduction of LV strain in childhood would predict a future decrease of LV ejection fraction in adult MFS patients.

In our study, we observed a borderline significant RV GLS attenuation in MFS patients concordant with the reduced RVEF. On the contrary, RV GCS was increased in MFS patients compared to controls. Currently, there is a gap of knowledge regarding RV strain in MFS patients, but there are some CMR studies on RV strain in patients with PE [19, 38]. In line with our results, Lollert et al reported on reduced RVEF, increased RV GCS and normal RV GLS in PE patients [38]. Truong et al also observed increased basal and apical RV circumferential strain in PE patients, but reduced values on mid-cavity [19]. Increased RV GCS might hint at a potential compensatory mechanism for RV GLS attenuation, but further strain studies are needed to verify this hypothesis.

## Limitations

Our study has several limitations. First, we had a small sample size. However, the number of studies reporting paediatric CMR data in general and strain analysis by feature-tracking CMR in particular is very limited and we believe that our study would add to the scientific knowledge of the research community. Second, this study was conducted with a retrospective design. Our CMR protocol included a short-axis cine stack in contrast to the only other CMR study by Winther et al reporting on adult MFS patients [9]. Third, we cannot provide any data on the influence of reduced LV strain on a possible development of LVEF reduction, which would require a long-term follow-up.

## Conclusions

This study investigated the effect of chest wall deformity in paediatric MFS patients on cardiac systolic function suggesting that increased Haller index may be associated with a decrease in RV volume, ejection fraction and longitudinal strain. Conversely, RV circumferential strain was increased in MFS patients, which may indicate a compensatory mechanism. Although MFS patients had normal LV volumes and ejection fraction, they demonstrated lower global LV longitudinal and circumferential strain compared to healthy controls. Our results are indicative of the existence of Marfan-related cardiomyopathy. Furthermore, follow-up studies are warranted to investigate the prognostic value of chest wall deformity and attenuated LV and RV strain in young MFS patients.

## Supplementary Information

ESM 1 (DOCX 31 kb)

## Abbreviations

BMI: Body mass index
BSA: Body surface area
BTFE: Balanced turbo field echo
CMR: Cardiovascular magnetic resonance
EDV: End-diastolic volume
EF: Ejection fraction
ESV: end-systolic volume
GCS: Global circumferential strain
GLS: Global longitudinal strain
GRS: Global radial strain
HI: Haller index
LA: Left atrial
LV: Left ventricle
MFS: Marfan syndrome
PE: Pectus excavatum
RA: Right atrial
RV: Right ventricle
SSFP: Steady-state free-precession
SV: Stroke volume
