# Supplementary material for: Impact of chest wall deformity on cardiac function by CMR and feature-tracking strain analysis in paediatric patients with Marfan syndrome
Source: Eur Radiol. 2020 Dec 23;31(6):3973–82. doi: 10.1007/s00330-020-07616-9 (PMC8128727; doi:10.1007/s00330-020-07616-9)
Supplement: Supplementary file 1 — (DOCX 31 kb) [file 330_2020_7616_MOESM1_ESM.docx]

**Supplemental Table E1:** Comparisons of all MFS patients without valvular disease and with valvular disease.

|  | **Without valvular disease (n = 28)** | **With valvular disease (n = 12)** | ***P* value** |
| --- | --- | --- | --- |
| **Demographics** | | | |
| Age, yrs | 16 ±3 | 16 ±3 | 0.78 |
| Male, % | 15 (54) | 9 (75) | 0.27 |
| Weight, kg | 62 ±19 | 61 ±24 | 0.88 |
| Height, m | 1.79 ±0.17 | 1.79 ±0.15 | 0.95 |
| BMI, kg/m^2^ | 19 ±4 | 19 ±4 | 0.74 |
| BSA, m² | 1.78 ±0.34 | 1.76 ±0.38 | 0.86 |
| **CMR parameters** | | | |
| Heart rate at CMR, bpm | 70 ±10 | 74 ±14 | 0.30 |
| LVEF, % | 61 ±5 | 62 ±4 | 0.40 |
| LV mass, g/m² | 53 ±11 | 59 ±16 | 0.17 |
| LVEDV, ml/m² | 83 ±21 | 93 ±14 | 0.16 |
| LVESV, ml/m² | 32 ±7 | 35 ±8 | 0.20 |
| LVSV, ml/m² | 49 ±11 | 58 ±8 | **0.02** |
| LAEDV, ml/m² | 13 ±6 | 18 ±9 | **0.04** |
| LAESV, ml/m² | 25 ±9 | 33 ±13 | **0.04** |
| RVEF, % | 53 ±10 | 61 ±7 | **0.02** |
| RVEDV, ml/m² | 76 ±18 | 74 ±14 | 0.76 |
| RVESV, ml/m² | 36 ±10 | 30 ±9 | 0.08 |
| RVSV, ml/m² | 40 ±13 | 44 ±8 | 0.30 |
| RAEDV, ml/m² | 18 ±5 | 20 ±9 | 0.42 |
| RAESV, ml/m² | 32 ±9 | 35 ±12 | 0.48 |
| **Chest wall dimensions** | | | |
| A-Haller, mm | 228 ±22 | 226 ±28 | 0.85 |
| C-Haller, mm | 74 ±24 | 83 ±27 | 0.30 |
| Haller index | 3.5 ±1.6 | 3.1 ±1.4 | 0.41 |
| **Global LV myocardial strain** | | | |
| LV GLS, % | -17 ±2 | -18 ±4 | 0.24 |
| LV GCS, % | -19 ±3 | -18 ±4 | 0.29 |
| LV GRS, % | 35 ±7 | 34 ±9 | 0.83 |
| **Global RV myocardial strain** | | | |
| RV GLS, % | -21 ±2 | -22 ±3 | 0.25 |
| RV GCS, % | -14 ±3 | -15 ±3 | 0.62 |

Numbers are mean ±SD for continuous and n (%) for categorical data.

**Abbreviations:** BMI, body mass index; BSA, body surface area; GCS, global circumferential strain; GLS, global longitudinal strain; GRS, global radial strain; HR, heart rate; LAEDV, left atrial end-diastolic volume; LAESV, left atrial end-systolic volume; LV, left ventricular; LVEF, left ventricular ejection fraction; LVEDV, left ventricular end-diastolic volume; LVESV, left ventricular end-systolic volume; LVSV, left ventricular stroke volume; RAEDV, right atrial end-diastolic volume; RAESV, right atrial end-systolic volume; RV, right ventricle; RVEDV, right ventricular end-diastolic volume; RVEF, right ventricular ejection fraction; RVESV, right ventricular end-systolic volume; RVSV, right ventricular stroke volume.
